# The construction of a conceptual framework explaining the relation between barriers to change of management of neuropsychiatric symptoms in nursing homes: a qualitative study using focus groups

**DOI:** 10.1186/s12877-020-01569-w

**Published:** 2020-05-06

**Authors:** Charlotte F. van Teunenbroek, Kim Verhagen, Martin Smalbrugge, Anke Persoon, Sytse U. Zuidema, Debby L. Gerritsen

**Affiliations:** 1 Department of General Practice and Elderly Care Medicine, University of Groningen, University Medical Center Groningen, PO Box 196, 9700 AD Groningen, HPC FA21, the Netherlands; 2 Department of General Practice and Elderly Care Medicine, Amsterdam Public Health research institute, Amsterdam University Medical Centers, location VUmc, Amsterdam, the Netherlands; 3 Radboud Institute for Health Sciences, Department of Primary and Community Care, Radboud Alzheimer Centre, Radboud University Medical Centre, Nijmegen, the Netherlands

**Keywords:** Barriers to change, Extent of change, Focus groups, Intercollegiate relations, Nursing homes, Quality improvement, Qualitative research

## Abstract

**Background:**

Several efforts have been made to change management of neuropsychiatric symptoms (NPS) in nursing homes, however only few were successful. Numerous barriers to change in healthcare were identified, yet only one conceptual model is known to study their interrelationships. Unfortunately, this model does not discuss specific barriers encountered in nursing home practice. The aim of this study is to explore perceived barriers to change in the management of NPS in nursing homes and to construct a conceptual framework providing insight into the relative importance and interrelationships of these barriers when improving quality of care.

**Methods:**

Four focus groups were conducted in different dementia special care units of one Dutch nursing home. Participants were either nursing staff, treatment staff or relatives of residents. Qualitative thematic analysis was conducted according to the five phases defined by Braun & Clarke. Finally, a conceptual framework showing the interrelations of barrier-themes was constructed using text fragments of the focus groups.

**Results:**

We constructed a conceptual framework consisting of eight themes of barriers explaining the extent to which change in NPS-management can be achieved: ‘organizational barriers’, ‘personal barriers’, ‘deficiency of staff knowledge’, ‘suboptimal communication’, ‘inadequate (multidisciplinary) collaboration’, ‘disorganization of processes’, ‘reactive coping’ and ‘differences in perception’. Addressing ‘organizational barriers’ and ‘deficiency of staff knowledge’ is a precondition for change. ‘Suboptimal communication’ and ‘inadequate (multidisciplinary) collaboration’ play a key role in the extent of change achieved via the themes ‘differences in perception’ and ‘disorganization of processes’. Furthermore, ‘personal barriers’ influence all themes - except ‘organizational barriers’ - and may cause ‘reactive coping’, which in turn may lead to ‘difficulties to structure processes’.

**Conclusions:**

A conceptual framework was created explaining the relationships between barriers towards achieving change focused on improving management of NPS in nursing homes. After this framework has been confirmed and refined in additional research, it can be used to study the interrelatedness of barriers to change, and to determine the importance of addressing them for achieving change in the provided care.

## Background

In the Netherlands 80.000 people with dementia reside in care facilities such as nursing homes [1]. Of these residents, approximately 27% use antipsychotic drugs as a treatment for neuropsychiatric symptoms (NPS) and 40% use antidepressants [2]. Guidelines advise a restricted use of psychotropic drugs in the treatment of NPS and advocate the use of psychosocial interventions [3]. The analysis and treatment of NPS is a multidisciplinary process, wherein, among others, the physician, psychologist and nursing staff play an important role [4]. Proper treatment of NPS is important, due to the negative influence of improper treatment of NPS on the quality of life of residents and on nursing staff. For example, nursing staff might experience anxiety and burnout as a result of NPS in residents [5, 6]. Therefore, various multidisciplinary interventions have been developed to reduce the frequency of psychotropic drug use and/or NPS, or to improve residents’ quality of life [4, 7–12]. Unfortunately, the (long-term) effectiveness of many of these interventions in terms of reduction of psychotropic drug use was shown to be limited [7, 8, 12, 13].

The small effects of interventions for psychotropic drug use may be a result of difficulties to implement interventions and induce change in nursing homes [14]. In that respect, a number of studies has been conducted to identify specific barriers towards implementation of (complex) interventions in nursing homes, often by means of a process evaluation. A major barrier – which has been reported on multiple occasions – is the complexity of the guideline or intervention to be implemented [15, 16]. These interventions are frequently complex due to a multidisciplinary approach, in which each discipline (i.e. nurses, physicians, and psychologists) applies different types of interventions [4]. In addition, a major barrier reported was the high turnover of the nursing home workforce [14–19]. Moreover, reorganizations, other innovations running at the time of the intervention, no relevance felt by the staff [8, 17, 18, 20], and the culture of the care unit - including attitude towards change -, are barriers towards changing current practice [14].

In the past, research has been conducted to identify barriers and to classify these into categories i.e., themes. For example, Mentes & Tripp-Reimer [19] provide an overview of barrier themes encountered in nursing home research: residents, staff, administrative and organizational issues, attitudes, research protocols and research assistants. Furthermore, Corazzini et al. [21] studied challenges (barriers) encountered while implementing ‘culture change in nursing home staff’ and ‘leadership behaviors’ that facilitated this change. They found six key themes, which described these challenges and leadership behaviors: ‘relationships’, ‘standards and expectations’, ‘motivation and vision’, ‘workload’, ‘respect of personhood’ and ‘physical environment’ [21]. Finally, the English National Institute for Health and Clinical Excellence (NHS) carried out a systematic review, which offered five types of barriers to change in healthcare and ideas to overcome these barriers. The five types of barriers identified were: ‘awareness and knowledge’, ‘motivation’, ‘practicalities’, ‘acceptance and beliefs’ and ‘skills’ [22]. Identifying themes of barriers is important as these can assist in understanding the causes of barriers and how to address these. Yet, insight into the relationships between themes of barriers is even more helpful in effectively addressing barriers, as it allows for a better determination of the magnitude of the barrier and of strategies to resolve it [23]. There is evidence indicating that assessment of barriers – before attempting implementation of an intervention or attempting to change current practice – will increase the chance of success [24].

In relation, Van Bokhoven et al. [23] mention a modified ‘model of barriers and facilitators’ based on the PRECEDE-PROCEED concept and theory of planned behavior, to provide a foundation for a structured quality improvement intervention. This model focuses especially on improvement of quality of life of residents and pertains to health care practice in general. However, the model does not include specific barriers encountered in nursing home practice, nor does it address the barriers encountered in improvement of the quality of care. Although many barriers to change and overarching themes have been identified in previous research, there is no framework available that explains the relationship between these perceived barriers in nursing homes. In a nursing home, the residents, professionals and provided care have specific characteristics that may result in encountering other barriers to change than in other settings, warranting a study that is specific for nursing homes [25]. Therefore, the aim of this study is to explore the perceived barriers to change regarding management of NPS in nursing homes and to construct a conceptual framework providing insight into the importance of and relationships between these barriers.

## Methods

### Design and setting

A pilot study was conducted in preparation of a larger trial ‘Reduction of Inappropriate psychotropic Drug use in nursing home patients with dementia’ (RID) [26]. In this pilot study, focus groups were formed to identify barriers to change in nursing homes. The focus group interviews took place in a Dutch nursing home in the time span of 1 week in March 2015, wherein all involved professionals were employed by this nursing home. Qualitative thematic analysis [27] was used to identify barriers to change and their interrelations. We complied with the COREQ checklist in conducting and reporting this study, see supplement A [28].

To illustrate the Dutch nursing home setting; in the Netherlands, a psychogeriatric unit of a nursing home has been built in either two ways to adapt to the needs of their residents with dementia: (1) a (traditionally built) larger scale care unit with multiple living rooms, interconnecting corridors and closed-door systems or (2) a small-scale living facility, wherein the unit resembles a general home, which is equipped with a kitchen, living room and individual bedrooms [25]. Life in these small-scale living facilities is meant to closely resemble normal life outside the nursing home. An elderly care physician is usually responsible for the medical treatment of the residents and is employed by the nursing home [29]. Nursing staff comprises different levels of nursing and education levels, the European Qualification Framework (EQF) provide a framework to describe involved nurses [30]. In addition to the elderly care physician, Nurse practitioners have their own medical responsibility and work in close cooperation with the attending elderly care physician to deliver medical treatment (EQF level 7) [31]. Licensed practical nurses (LPN, EQF level 3) work independently in providing care and are responsible for their individual actions and practice. Their responsibilities entail the day to day care of residents (including handing out medication), supervising other nursing staff and assessing the residents’ physical and mental health [32]. When an LPN has attended additional education, he or she can function as the responsible LPN (RLPN) for the coordination of care for individual residents and their proxy (educational level EQF level 3). Nurse assistants (NA; EQF level 2) are responsible for the day to day care of residents (excluding handing out medication). Lastly, all Dutch facilities employ several disciplines to improve the wellbeing, functioning and quality of life of residents: psychologists, physiotherapists, occupational therapists, dietitians, speech therapists and an activity therapist or activity assistant.

**Fig. 1:**
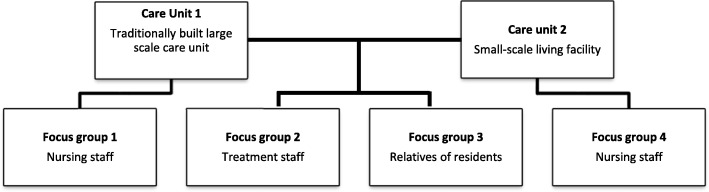
Distribution of focus groups

Four (monodisciplinary) focus group discussions were organized in two care units of one nursing home in the Northern part of the Netherlands. To increase diversity of the sample, one traditionally built large scale care unit and a small-scale living facility were included in this research. Two focus groups included nursing staff and their manager (group 1 & 4), one included only treatment staff (group 2) and one relatives (group 3), see Fig. 1. The nursing staff was recruited via the unit managers. The treatment staff and relatives were recruited by the principal researcher (SUZ) and the unit managers. Staff was approached face-to-face for participation, relatives of residents were approached via mail.

### Data collection

Participants of the focus groups were stimulated to express their views and exchange opinions on difficulties in the care process of their care unit for residents with dementia, with a specific focus on NPS and psychotropic drug use. Furthermore, participants were stimulated to discuss general barriers concerning possible implementation of interventions to address and improve the treatment of NPS and reduce psychotropic drug use. A guide to direct the discussion was developed, based upon literature and consultation of clinical experts, following guidelines for conducting focus groups [33]. The focus group discussions were moderated by a psychologist from another location of the same care organization. To prompt statements on barriers, questions were asked about one or more of the following practical topics: [1] mutual expectations on collaboration among members of the nursing staff, unit manager, physician, psychologist, other disciplines and relatives to detect, diagnose and treat residents with NPS, [2] the actual use of the Dutch guideline for problem behavior (3), [3] the applied work plan for signaling NPS, [4] knowledge about residents’ background, [5] applied treatment solutions for NPS, [6] knowledge and experience of various disciplines, [7] reasons for prescribing psychotropic drugs and [8] limitations experienced in the management of NPS/psychotropic drug use. Interviews were audio-taped. Information on sex and profession of the participants was obtained.

### Data analysis

All interviews were transcribed ad verbatim, and transcriptions were cross-checked with the recordings afterwards. Qualitative thematic analysis was used by continued open coding, wherein barrier-themes identified in previous research were used as background information. Furthermore, the framework was refined until no new information could be added from the existing data gathered in the four focus groups, and the stage of conceptual saturation was reached [27]. The ultimate goal was the construction of a model to identify connected topics [34].

Data analysis was an iterative process according to the five phases described by Braun & Clarke (2006) and was conducted by two researchers (C.T. and K.V.). C.T. has a background in medicine, while K.V. has a background in psychology. The researchers started the analysis by reading and familiarizing with the data (phase one: familiarizing yourself with your data). Hereafter, relevant quotations for answering the research question were independently marked as free quotations using Atlas.ti software v 7.5.10, (Atlas.ti Scientific Software development GmbH, Berlin, Germany). Next, the researchers individually labelled these quotations with codes, staying as close to the text as possible. In addition, memos were attached to contradictions and deviating opinions that were observed by the researchers between or within the transcripts of the focus groups. Then, the researchers discussed all codes until consensus was reached (phase two: generating initial codes). Subsequently, both researchers independently categorized all codes into barrier-subthemes (using ‘clustered codes’ and ‘subthemes’ in Atlas.ti) and discussed these until consensus was reached to ensure reliability. Afterwards, the researchers (C.T. and K.V.) had multiple meetings to analyze and discuss the relation between different barrier-subthemes. Barrier-subthemes that were related, were brought together in themes of barriers (themes) by D.G. and C.T. (phase three and four: searching for themes; reviewing themes). In addition, all memos were crosschecked with the identified themes to check for new insights and content that was not in line with comments from individual participants. Memos with the same content were categorized together and included in the analysis. After grouping all barrier-subthemes into themes, themes were named according to their content (phase five: defining and naming themes). The interrelations between themes of barriers were defined by using text fragments of the focus groups and hereafter visualized in a conceptual framework. Four researchers (C.T., K.V., D.G. and A.P.) had multiple discussions to construct this framework.

### Ethical approval

The study was undertaken in accordance with the declaration of Helsinki [35], the applicable Dutch legislation and in agreement with the code of conduct of Health Research [36]. It has been assessed by the Institutional Review Board of the University Medical Center Groningen (UCMG), which stated that no approval was needed as this non-invasive study was not subject to the Dutch Medical Research Involving Human Subjects Act (METC decision: METc 2014/405). All participants of the focus groups have consented to the participation in and audiotaping of the interviews. Verbal informed consent was recorded on tape. The interviews were transcribed and analyzed with anonymized codes.

## Results

### Participant characteristics

Four focus groups were conducted, see Fig. 1. Focus groups 1 and 4 consisted of nursing staff, all female with different levels of education [30]. Focus group 1 encompassed LPN (*N* = 2; EQF level 3), RLPN (*N* = 1, EQF level 3), and the unit manager (UM; *N* = 1; physiotherapist) of the care unit. Focus group 4 consisted of the following participants: LPN (*N* = 2, EQF level 3), RLPN (*N* = 2, EQF level 3), NA (*N* = 2; EQF level 2) and UM (*N* = 1; registered nurse; EQF level 6). The focus group of treatment staff consisted of: registered nurses who are responsible for behavioral treatment decisions outside office hours (RN; *N* = 2), psychologists (P; *N* = 2), a nurse practitioner who functions at the level of a physician (NP; *N* = 1, EQF level 7) and a behavioral coach who is responsible for behavioral treatment decisions within office hours (BC; *N* = 1), one of whom was male. The last focus group consisted of four partners and two adult children of residents. Half of the relatives was female, half was male. The focus groups took between 84 and 115 min. In the results presented below, the word ‘participants’ is used when participants of all four focus groups reported these findings, in any other case the participant’s function is mentioned.

### Thematic analysis

The analysis resulted in the identification of eight themes of barriers: ‘Organizational barriers’, ‘Personal barriers’, ‘Deficiency of staff knowledge’, ‘Inadequate (multidisciplinary) collaboration’, ‘Suboptimal communication’, ‘Disorganization of processes’, ‘Reactive coping & resilience of organization’ and ‘Differences in perception’. These interacting themes of barriers were brought together in a conceptual framework explaining the extent to which change regarding management of NPS is impaired in a nursing home, given the existing barriers. Some of these barriers are explicitly linked to prohibiting change, as shown in corresponding quotations, others regard impediments to good care, indirectly impairing change. Firstly, we will describe the barrier-subthemes and themes: the building blocks of which the framework is composed. Thereafter, the conceptual framework, which shows the relationships between the themes, will be described.

Additional quotations to the ones mentioned in the results below, are included in Table 1 (appendix). Each quotation is addressed by its corresponding code: the letter corresponds with the theme, the number with the quotation within that theme, i.e. A1, H5.

#### Organizational barriers

The first theme consists of barriers that were related to the organization and organizational decisions. This theme is composed of the following subthemes: ‘Use of temporary staff’, ‘Insufficient staff on the unit’, ‘discontinuity by frequent staff turnover’, ‘Lack of time’ and ‘Lack of continuous education’. The ‘use of temporary staff’ and a ‘lack of sufficient staff’ on the unit (A4) inhibited the implementation of interventions as well as the continuity of care (A1). In addition, a difficulty in maintaining the continuity of care was caused by ‘turnover’ within the ranks of the physicians (A13) and a ‘turnover’ within the nursing staff (A7, A12). Furthermore, these barriers impeded the extent of change reached.

*A11, “We have actually had many different physicians here the past year, now another new one. And every physician also has their own method. And own mindset. And has their own vision on this [psychotropic drug prescription]. And we have to change.”****RLPN (pa22)***

Moreover, a lack of time influenced the transfer and consistency of information between nursing staff (A16) and has been mentioned by the psychologist to impair the information extraction about residents (A17). Lastly, participants indicated that continuous (cyclic) training for nursing home staff was important to get inspired, acquire new insights, and to incorporate these insights into daily practice (A18). The absence of continuous (cyclic) training is a barrier to change.

#### Personal barriers

The second theme consists of barriers that are related to personal factors of staff members and relatives. This theme is composed of the following subthemes: ‘Reduced staff motivation and effort’, ‘Negative staff emotions’ and ‘Dissatisfaction of relatives’. Participants stressed differences in ‘reduced staff motivation and effort’ among staff members. It was considered important to show motivation by showing effort to gain more knowledge, for example on diseases, but participants mentioned that others did not.

*B1: “It’s also up to the person, I think. One is interested more quickly, as you said yourself, to search themselves, what fits with this disease, what should I think of? Is there another approach necessary? Someone else might think: Do I care? I work here and that’s it. { … } I think there are a lot of differences between colleagues.****RLPN (pa4)***

*One will deepen their knowledge more than others.”****RLPN (pa4)***

Furthermore, another important barrier-subtheme within the theme personal barriers was ‘negative staff emotions’. It primarily entailed hopelessness of nursing staff regarding the interaction with residents or treatment staff and the proposed treatment of behavior (B4-B6).

Lastly, the ‘dissatisfaction of relatives’ might negatively influence the amount of change possible, through repeatedly expressing their disappointment in the matters at hand. In particular, dissatisfaction of relatives was apparent when problems arose on the unit with their relative. Relatives sometimes felt disappointed about turnover of staff and temporary workers (B7).

#### Deficiency of staff knowledge

The third theme consists of barriers that are related to knowledge and has no subthemes. The treatment of NPS and therefore also prescription of psychotropic drugs was strongly related to knowledge of staff, or a deficiency thereof (C1).

*C3, “And if someone totally panics because he sees big spiders walking on the wall, then you know … . Oh … that fits the picture of the disease. So, he sees things that are not there. You can panic about that and so yes … as long … if you don’t have that knowledge … then you would think … that man is not well at all. I have to call the physician quickly as he has to go to the hospital.”****LPN (pa3)***

#### Inadequate (multidisciplinary) collaboration

The fourth theme consists of barriers that are related to inadequate (multidisciplinary) collaboration. This theme is composed of the following subthemes: ‘Lack of evaluation’, ‘Lack of (multidisciplinary) consultation of key disciplines’ and ‘Lack of multidisciplinary consultations / meetings’. The participants indicated that lack of evaluations of initiated processes of change and of treatments started was a key barrier in inadequate (multidisciplinary) collaboration.

*D1, “In past several years, if someone was given a physical restraint, then that usually remained that way. And before it comes up for discussion again or before it gets discussed like ‘is it actually still necessary that someone is restrained’, that woman is not going to get up anymore. That you … If no one makes a remark about it, that sometimes persists longer than necessary.”****BC (pa10)***

Additionally, not consulting other key staff members, such as RN, LPN and RLPN, impaired a healthy (multidisciplinary) collaboration, even though the exclusion of these members was not done consciously (D3). Lastly, the lack of frequent meetings with this staff was considered odd and might have impaired the establishment of new and effective treatments for residents (D5).

#### Suboptimal communication

The fifth theme consists of barriers that are related to communication. This theme is composed of the following subthemes: ‘Flawed internal reporting and communication’, ‘Lack of sharing experiences’, ‘Unclear communication of changes with family’ and ‘Communication with relatives is considered time consuming’. The theme ‘suboptimal communication’ is a very broad theme, entailing different kinds of aspects such as: 1) communication between staff as seen by relatives, 2) communication between staff as seen by the staff and 3) communication between relatives and staff as seen by staff and relatives.

One of the relatives of a resident described the communication between nursing staff members as flawed, impairing the process of care (E1). In addition, one of the psychologists mentioned he does not always communicate with his colleagues on who will communicate with the relative (E3). Participants stated ‘lack of sharing experiences’, such as asking for help and sharing success stories, was important to inspire each other into improving care, whereas lack thereof was seen as a barrier.

*E5, “Especially the old school [LPN], they really have a … really a … a culture of wanting to control, they want to have the right touch. And if they need to ask for help, sometimes that is a … that is too much to ask. Or a … Or … One is not so easily inclined to share a problem. They keep it to themselves. And I find that very unfortunate.”****BC (pa10)***

In addition, there was confusion about the communication of alternations (for example in medication) with family. The physician expected nursing staff to discuss certain alternations in medication with relatives, while the nursing staff experienced difficulties explaining these to the relatives due to flawed reporting by the physician in the patient file (E7). Furthermore, an LPN remarked she thought the communication about the resident with relatives was time consuming. Therefore, often only the bare essentials about the resident were discussed. This resulted in incomplete information in the patient file (E9).

#### Disorganization of processes

The sixth theme consists of barriers that are related to disorganization of processes. This theme is composed of the following subthemes: ‘Unstructured processes’, ‘Ambiguity of the division of responsibilities and tasks’ and ‘Decision-making culture’. This theme entailed information related to the obstacles, either culture-based or related to a key person, in organizing (care) processes. The necessity of structuring evaluation and consultation about NPS and its treatment was primarily mentioned by the nurse practitioner and psychologists (F1, F3). Furthermore, obstacles in structuring processes were mentioned, such as ideas that do not converge (F4). Moreover, participants expressed confusion concerning the division of responsibilities and tasks. Especially ambiguity about the person who manages the process of care was mentioned (F9, F11).

*F6, “I think it is important, that they [physician and psychologist] are in a position … in which they can collaborate. So that it is clear, who does which task? Eh … Who is the coordinator? Is the physician the main point of contact in case of NPS or is it the nurse practitioner? Or is it the psychologist? I sometimes find that difficult, I sometimes think who is the captain on that ship?”****P (pa6)***

Lastly, within this theme, the ‘unfulfilled expectations of management’ and their support of staff are important barriers. Staff expected the unit manager to coach and inspire the nursing staff, while in practice the unit managers were predominantly busy with planning tasks (D7).

The last item mentioned in this theme was the culture of trying to reach consensus when making a decision. This culture was seen as frustrating by participants, which elongated the time necessary to structure processes (F12).

#### Reactive coping & resilience of organization

The seventh theme consists of barriers that are related to resilience of the organization or reactive coping of the persons within that organization. Reactive coping is a coping style in which one awaits circumstances to unfold before responding, which may complicate initiation or maintenance of change. This theme is composed of the following subthemes: ‘Difficulty breaking patterns’, ‘Concerns relatives on changing practice’, ‘Responding late to behavior’ and ‘Not signaling changes in behavior’. Participants mentioned how difficult it was to change existing practice and that sometimes they encountered resistance (G2, G3). The manager of one of the care units explained that it is difficult to break existing patterns, to change.

*G1, “ … things that are going like this for years, yes that is very hard to break through, to change. That is in everything on this care unit.”****UM (pa1)***

Furthermore, the organization did not proactively involve the relatives in the decision process. Relatives voiced their concerns about the way their input about the care of their relative was not used in the nursing home. They said they did not have any influence on the care process (G4) and that although the relatives were sometimes consulted by the nursing staff, this consultation took place after the final decision already had been made (G5).

In addition, an LPN mentioned a tardiness in responding to behavior of residents by involving other disciplines afterwards, when the damage was already done (G10). Although interventions have been used to improve the timing, nursing staff maintained their behavior of delayed responding. ‘Responding late to behavior’ and ‘Not signaling changes in behavior’ by staff impaired the care process (G11).

#### Differences in perception

The eighth theme consists of barriers that are related to differences in perception. This theme is composed of the following subthemes: ‘Expressed differences in perception between colleagues’ and ‘Observed differences in perception between focus group participants’. The first subtheme was mentioned by participants in the focus groups, while the second was observed in the transcripts between and within the different focus groups by the researchers. These two subthemes are a broad collection of all differences and controversial views expressed and observed in the focus groups.

There were two ways by which the ‘expressed differences in perception between colleagues’ became clear. First, the participants mentioned differences in the experience of norms and values (H1), vision and work approach and attitude between colleagues (H2, H3). Secondly, there was a difference in view on the course of affairs on for example evaluations by physicians/psychologists and care staff**,** as was illustrated by the psychologist and nurse practitioner.

*H4, “I think those* [restrictions of freedom of the resident] *are being evaluated by the physician in the rounds, monthly. That’s not something that’s discussed multidisciplinary … ”***P (pa6)**

*If I’m honest, I have never experienced that* [evaluation of restrictions of freedom of the resident] *before.”****NP (pa7)***

These quotes show that the different disciplines were not aware of the activities, work and tasks of the other. In addition, several differences in perception between focus group participants were observed by the researchers, while transcribing and analyzing the data, using memos. The psychologist mentioned he did not see any need in the presence of registered nurses in the multidisciplinary meetings about behavior of residents, while later on in the same focus group, the nurses emphasized it would have been useful for them to be present in such meetings.

*H5, “People are broadly discussed in the multidisciplinary meetings. There we address what they need … { … } What would be good interventions, fitting for that person. So, then we have a much broader context than … where we talk about someone. Of course, not everyone is present. For example, you [registered nurses] do not have anything to do with that.”****P (pa5)***

*H6, We are actually never present at such meetings [multidisciplinary consultation]. { … } It would be relevant if we’d be present there. Because we work in the evenings, we work at night, the weekends. We are here such a big part of the time. We are always the ones that get called .”****RN (pa8)***

Furthermore, the nurse practitioner thought nursing staff informed relatives about changes in medication. However, nursing staff were under the impression that the nurse practitioner or physician would inform the relatives (H9, H10). Another contradiction was observed about the assumptions on necessity to structure meetings between a unit manager and behavioral coach/nurse practitioner. The unit manager did not want to structure the frequency of evaluation meetings; according to her, this was not necessary in a small setting. The other group, however, emphasized that structuring the frequency and time of these meetings would improve the continuity of care., because the meetings often didn’t take place (H7, H8).

Moreover, the staff remarked that relatives had little complaints, while relatives mentioned many complaints in their focus group, for example on staff turnover (H11, H12).

### Relationship and hierarchy between barrier-themes

**Fig. 2:**
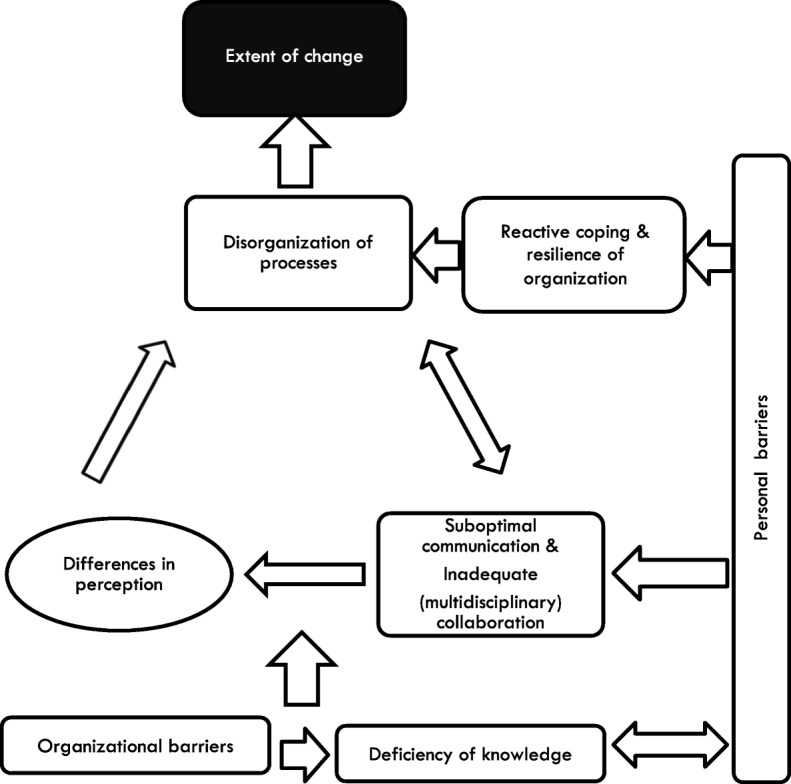
Framework depicting relations between themes to explain the extent of change (black box). The round box depicts that this theme is mentioned by participants as well as observed within transcripts between and within focus groups through memos

Next, based on the accounts of the participants and our observations in the transcripts of the focus groups, we will explain the relations and hierarchy between the different themes by means of a conceptual framework (see Fig. 2).

Figure 2 starts at the bottom with the themes ‘Organizational barriers’ and ‘Deficiency of staff knowledge’. Participants mentioned ‘Organizational barriers’ (especially turnover and temporary staff) in relation to all mentioned themes above, making this theme one of the starting points for the possible hindrance of change. On the same level, we identified the theme ‘Deficiency of staff knowledge’, which was directly influenced by ‘Organizational barriers’; participants mentioned that a ‘lack of time’, ‘discontinuity by frequent staff turnover’ and the ‘use of temporary staff’ in itself created a deficiency of knowledge in the unit. One of the relatives described the phenomenon of ‘temporary staff’ as follows: *“They are appointed by the employment agency, well … nine out of ten times, they do not know chalk from cheese.”***FM (pa14).**

The third layer consists of an interaction between the themes ‘Suboptimal communication’ and ‘Inadequate (multidisciplinary) collaboration’, ‘Differences in perception’ and ‘Disorganization of processes’. ‘Suboptimal communication and ‘Inadequate (multidisciplinary) collaboration’ were so strongly related that they were put in the same box, there was no way to say which of these themes influenced the other. A poor quality of communication impeded good collaboration and sharing of information, which disrupted structuring of processes. The following was said about this relation: *“I think it is important, that they [physician and psychologist] are in a position … in which they can collaborate. So that it is clear, who does which task? Eh … Who is the coordinator? Is the physician the main point of contact in case of NPS or is it the nurse practitioner? Or is it the psychologist?”***P (pa6).**

‘Suboptimal communication’ and ‘Inadequate (multidisciplinary) collaboration’ were causes for observed discrepancies in perception and assumptions. These observed discrepancies in perceptions and assumptions led to unstructured processes, according to the participants (F7 & F8, F9 & F10, G3). There was no structured approach and there were many ambiguities about agreements made (G7 – G10). Moreover, the unstructured approach and ambiguous agreements resulted in impediments for a structured collaboration and structured deliberations on NPS.

Next, there were two relations: first, ‘Personal barriers’ separately enhanced the negative influence of ‘Reactive coping & resilience of organization’, which was strongly related to ‘Disorganization of processes’ and, through that theme, to the extent of change. Second, an interaction was present between ‘personal barriers’ and ‘disorganization of processes’, via ‘reactive coping’. When staff motivation and effort was reduced, there was usually a reactive coping style, inhibiting the start of structuring processes. In their turn, the subsequent difficulties which can be encountered, caused a reactive coping style and frustration (negative emotions) in staff.

*“But, again, today I encountered that the behavioral coach wasn’t contacted. So, I think that’s very frustrating.”****NP (pa7)***

It was difficult for care professionals to break already existing behavioral patterns and try a new approach, which impeded collaboration to structure processes (H5, H7). ‘Personal barriers’ were related to all themes except organizational barriers. They were strongly related to the theme ‘deficiency of staff knowledge’, since a ‘reduced staff motivation and effort’ lead to a decrease in knowledge of staff (B2). Furthermore, regarding the identified barriers ‘reduced staff motivation and effort’, ‘suboptimal communication’ and ‘inadequate (multidisciplinary) collaboration, some participants explained that communication and collaboration were a result of motivation and effort of staff. ’.

*“I’m always a little bit earlier, you [other LPN] always come a little earlier too, so you’ll sit down or leave later. That facilitates information exchange.***RLPN (pa23)**

*Because I just joined the team, I think it’s very important for me to receive more information. Obviously, you read, but it is more pleasant to consult like this [face-to-face]. So sometimes I stay a little bit longer.”***LPN (pa19)**

Finally, the result of all previously mentioned themes of barriers, seemed to influence the extent to which change of care processes was impaired in the nursing home.

## Discussion

In this study we focused on the identification of perceived barriers to change in nursing homes and we aimed to construct a conceptual framework explaining the relation between these different barriers. We extracted eight themes of barriers that impede the extent to which change is likely. Some are direct barriers and some are indirect barriers. For example, ‘suboptimal communication’, ‘inadequate (multidisciplinary) collaboration’ and ‘reactive coping & resilience of organization’ are indirect barriers. These themes do not necessarily influence the extent of change directly, but do so via another theme or route. All identified themes are hierarchically related, wherein ‘organizational barriers and ‘deficiency of staff knowledge’ were the foundation of all other themes. Hereafter, ‘suboptimal communication’ and ‘inadequate (multidisciplinary) collaboration’ may cause ‘differences in perception’, which in turn can lead to disorganization. In addition, ‘personal barriers’ may influence ‘reactive coping & resilience of organization’ and via that route influence ‘disorganization of processes’. Moreover, ‘personal barriers’ influence these interacting layers. Especially ‘reduced staff motivation and effort’ and ‘negative staff emotions’ play an important role herein. The extent to which possible change is impaired can be determined by identifying existing barriers and categorizing them according to the conceptual framework. Subsequently addressing these barriers could enhance the possibility to change.

Various barriers, found in this research, are known from previous research. For example, ‘organizational barriers’, ‘personal barriers’ (such as a reduced motivation and effort) and ‘deficiency of staff knowledge’ are well-known categories of barriers to change [8, 14–22]. Our study adds that these categories may be the fundament to achieving change; without proper knowledge, organizational support and personal factors there will only be a small extent of change possible. Similarly, Zwijsen et al. [14] and the National Institute for Clinical Studies [37], among others, have identified issues in ‘inadequate (multidisciplinary) collaboration’ and ‘suboptimal communication’ before. We found two additional interrelated themes that influenced the possibility of impaired change, which were not identified before; ‘Differences in perception’ and ‘Reactive coping & resilience of organization’.

Although many studies have identified themes of barriers, only one elaborated on the relations between the different themes [23]. Whereas van Bokhoven et al. [23] constructed a framework wherein the barriers are split into external factors and professional factors influencing and explaining professional behavior, our framework focuses on the explanation of the extent to which change can be hindered. Due to the similar organizational nature of nursing homes and the fact that we recruited participants from both a small-scale living facility and a large scale care unit, this framework might be transferable to other nursing homes. However, first our framework should be confirmed and refined in additional research. Thereafter, our framework could be a tool for classifying barriers and identifying which problems might arise in the process of change. Some researchers have already tried to take ‘known barriers to implementation’ into account when implementing an intervention [38, 39]. Others actually identified the local barriers towards implementation before starting the implementation, to allow for optimal implementation of interventions [40]. Furthermore, approaches are available to assess the readiness to change in an organization, among others ‘the nursing home working conditions survey’ [41, 42]. Future implementation research could focus on identifying local barriers and classifying them with our framework to allow assessing the impact on the extent of change. After classification, a specific strategy for implementation could be chosen to enhance the effectiveness of implementation.

Some of the major studies included nursing staff and treatment staff in their focus groups [16, 21], acknowledging the importance of the influence of relatives and their perception [20]. Our study is one of the first to include family members in the focus groups to allow for a 360 degrees view of the barriers to change experienced in a nursing home. Furthermore, we underlined the importance of including nursing staff in the focus groups, because they form the bridge between treatment staff and patients and their relatives.

Although our study resulted in a novel framework explaining the relationships between barriers to change in nursing homes, it had some possible drawbacks. First, the study was carried out in preparation of selecting and implementing an intervention for reducing inappropriate psychotropic drug use. The focus of the focus group questions was therefore on management and treatment of NPS in combination with the prescription of psychotropic drugs. We asked concrete questions about suboptimal care and did not use the more abstract terminology of barriers to change. Due to this strategy we hope to have facilitated the conversation and to have elicited specific information about everyday practice. However, there is a possibility that we missed some of the barriers encountered. Secondly, the fact that the manager invited focus group participants and was present in the focus groups of the nursing staff and that the moderator sometimes asked provoking questions, could have negatively influenced participants to speak frankly. Next, there was no physician present in the focus groups since the attending physician was newly employed in this nursing home at the time of the research and was therefore unable to reflect on processes and change in this nursing home. Yet, a nurse practitioner functioning at the level of a physician was present, as was described in our methods section. Nevertheless, although many barriers mentioned concern actions of the physician, it is uncertain if the physician or nurse practitioner was meant. This might lead to a skewed interpretation of barriers. Finally, some barriers found in other research did not emerge in the focus groups in this study, such as culture on the care unit and complexity of the change or intervention trying to be achieved [14–16]. This might be a result of exploring barriers independent from implementing an intervention, including a solitary nursing home, not being able to work according to the principle of data saturation or simply a difference in perspective on the definition of the barrier. The two latter aspects are limitations to this study implying that it is too early to generalize the results. Nevertheless, it prompts investigation whether culture on the unit should be added to the model or whether it is reflected in barriers already present in the model, such as the ‘organizational barriers’, ‘inadequate (multidisciplinary) collaboration’ and ‘personal barriers’. Therefore, we suggest to broaden the scope to other nursing homes and to look into all barriers encountered in nursing home research, not only barriers related to NPS and psychotropic drugs use. Furthermore, we suggest to repeat our method of organizing different mono-disciplinary focus groups and analyze the data deductively, according to our framework, next to performing inductive analyses. In this way it can be assessed if our conceptual framework is complete or if some other (known) barriers or themes arise during the new analysis, complementing the framework. Lastly, we suggest research into facilitators to change. Although it is possible that the facilitators are the opposite of the barriers found, there is no certainty on these findings yet. This will result in a more complete picture of the possible extent to change in nursing homes and will provide practitioners with tools to implement changes and overcome barriers.

## Conclusions

We can conclude that we have provided a basic, conceptual framework explaining the relationships between different overarching themes of barriers towards achieving change in management of NPS in nursing homes. The framework may be used as a fundament to assess and to classify barriers to change after it has been confirmed and refined in additional research. It can assist in future research in the determination of steps to be taken when wanting to either improve the extent of change possible, or to establish the current extent to which change may be hindered. Future research could focus on the classification of local barriers and try to resolve and address these barriers. Specifically, the ranks of suboptimal communication, inadequate (multidisciplinary) collaboration and personal barriers call for action into resolving the barriers before attempting implementation of an intervention, to provide optimal implementation.

### Supplementary information

**Additional file 1: Supplement A.** Detailed applied methodology following the consolidated criteria for reporting qualitative studies (COREQ) 32-item checklist

## Data Availability

Authors can confirm that the datasets (transcripts of the interviews) used and/or analyzed during the current study are available from the corresponding author on reasonable request to ensure privacy and safety of the participants.

## Appendix

**Table 1:** Overview of all themes reflecting the mentioned barriers by participants, barrier-subthemes supporting these themes and relevant secondary quotes grounding the barrier-subthemes

Themes of barriers	Supporting barrier-subthemes	Relevant quotes
A. **Organizational barriers**		
	Use of temporary staff	A1, pa7: “Well I expect, if I allow to notify the behavior coach when there is agitation that they [LPN] will do so. But again, today I noticed that the behavioral coach wasn’t contacted. So, I think that’s very frustrating. Part of the reason was probably that there were temporary staff present.” **NP**
		A2, pa8:” Especially the regular staff, they show more commitment. {…} Especially the people from the employment agency, we have seen that before. (…) One time one of the temporary staff on the unit, a resident was showing agitation, but she ignored it completely and just walked past the resident.” **N**
		A3, pa20: “There are some things that remain undone when temporary staff is present. You just have to, as part of the team, you have to be aware of that.” **LPN**
	Insufficient staff on the unit	A4, pa8: “One evening, there were two (LPN’s) from the employment agency and there was one nurse aid present and the capacity was minimal. {…}.” **N**
		A5, pa2: “You know, of course you want to please every resident and everyone … but that’s not always possible. Or that you’re with too little staff …” **LPN**
		A6, pa2: “at the end of the week, oooh, there was too few staff, so you’re alone, from seven in the morning on.” **LPN**
	Discontinuity by frequent staff turnover	A7, pa18: “Relatives sometimes have complaints about us, that there is a lot of staff turnover, that happens on a regular basis. **NA**
		Pa17: Yes, that is the only complaint they have. **UM**
		Pa18: Fortunately!” **NA** A8, pa8: “With constantly changing staff, especially the physicians, you get a very ad hoc approach. And that is how it goes, because if one is present very little or … not present fulltime, there is someone different every time, you have to keep making new agreements.” **P**
		A9, pa2: “But of course you also have the turnover of the physicians. **LPN**
		Pa3 & pa4: Yes. **LPN & RLPN**
		Pa2: We have had many changes and every physician wants something else with it [psychotropic drugs] and sometimes I really think that is a disadvantage. **LPN**
		Pa4: Yes that is true. **RLPN**
		Pa2: We have had … how many physicians did we have in the past several years? **LPN**
		Pa4: I think I have worked with six.” **RLPN**
		A10, pa11: “The physicians also change often here.. **FM**
		Pa13: Yes the physician that was here now, that one has been here for a few months, but she already left again. **FM**
		Pa11: Yes … gone again. **FM**
		Pa13: I do not want to imply that this one isn’t good, that is not what I am trying to say. **FM**
		I: But it [the physicians] changes a lot? We just mentioned the regular team, but that also applies for the others?
		Pa15: My husband has been here for two years now, this is his sixth physician.” **FM**
		A11, pa22: “We have actually had many different physicians here the past year, now another new one. And every physician also has their own method. And own mindset. And has their own vision on this [psychotropic drug prescription]. And we have to change.” **RLPN**
		A12, I: You all actually indicate that staff varies strongly. With one you have a good connection and with the other you don’t.
		All: Yes … Yes … **FM**
		Pa13: I think that is the biggest mistake, the residents get very restless of all those unfamiliar faces. **FM**
		Pa 12 &15: Yes.. **FM**
		I: Exactly, so you notice a lot of staff turnover?
		Pa11: Yes, a lot.” **FM**
		A13, pa5: “To evaluate your actions is important. **P**
		Pa7: Also, with each other I think, how are we doing now? **NP**
		Pa5: To do that in a more structured way, that is our intention. **P**
		Pa5: And that actually works better with a regular team than if you have a changing team, because then … well … That needs no explanation.” **P**
		A14, pa15: “What we see now … Today that person is here, tomorrow it’s someone else, but that person doesn’t see that he [the resident] is behaving totally different from the day before or from usual. **FM**
		A15, pa17: “The thing is that … discontinuity of staff, that is … really a trigger for … eh … for behavior. Behavioral problems. That is … People just react to that. It is a trigger for mistakes also. But it is also {…} very important.” **UM**
	Lack of time	A16, pa17: “I heard in the work meetings for example was that the staff has little time to consult each other. There is no time to convey the information from shift to shift.” **UM**
		A17, pa5: “To extract the life history in clinical practice is found hard to execute. {…} There is a need … That is expressed by everyone. But the execution and time, that is a major problem.” **P**
	Lack of continuous education	A18, pa6: “But there should actually be cycles of training with pointers to deal with difficult behavior [of residents], there are new insights, we can inspire each other with cases from the past half year. Ehm … Then you’ll keep the spirits up together, you share, but that just does not get established. {…} There is not a kind of cycle like every few months there is a training for every team. That has to be stated [to management] every time again, that that is important.” **P**
B. **Personal barriers**		
	Reduced staff motivation and effort	B1, pa4: “It’s also up to the person, I think. One is interested more quickly, as you said yourself, to search themselves, what fits with this disease, what should I think of? Is there another approach necessary? Someone else might think: Do I care? I work here and that’s it. {…} I think there is a big difference between colleagues. **RLPN**
		Pa2: I think so too. **LPN**
		Pa4: One will deepen their knowledge more than others.” **RLPN**
		B2, pa7: “Well, I have to be honest, well … I have the idea that we are already heading the right direction if I’m very honest. Yeah … It can always be better, but that … you have to keep striving for that, but I think that the people who are here, that everybody is consciously working on that [improving prescription of psychotropic drugs]. And … yeah, so in that way we are already heading in the right direction. **NP**
		I: You emphasize the process that takes place in your team, you are very enthusiastic about the collaboration and the team, in a very broad sense?
		Pa7: Well the people that are here right now, I just notice, yeah, I also speak for myself, but I notice that everyone is benevolent to do and also to … to commit themselves to it. And is motivated for it. Yes … I really noticed that.” **NP**
	Negative staff emotions	B3, pa6: “I thought they [staff] were much more resistant. {…} They themselves became agitated. And to the person, or the behavior, they judged that, they judged the person and not the disease or the behavior that stemmed from that disease.” **P**
		B4, I: “How are NPS perceived?
		Pa6: Yes … well with irritation and also the feeling that there’s not much to do about it. There’s no point anyway. Or it will not get better.” **P**
		B5, I: “How do you experience NPS?
		Pa3: Sometimes like helplessness. Like there is nothing you can do about it. Sometimes this is what I think … then you’re really at a loss what to do.” **LPN**
		B6, I: “What do you expect of the physician in general?
		Pa22: I do not agree with it [psychotropic drugs] being stopped. Really, I do not agree with it. **RLPN**
		Pa19: Me neither. **LPN**
		Pa22: We are here all days, if you read [the report] you can see it. You [other LPN] are in the nightshift, you also know it. I really don’t agree with it, but whether I like it or not, it will happen. If the physician decides it … **RLPN**
		Pa19: Yes, then we don’t have a leg to stand on. **LPN**
		Pa22: I cannot influence it. **RLPN**
		Pa19: No … I find that disappointing.” **LPN**
	Dissatisfaction of relatives	B7, pa13: “There is not enough attention; people here already said it before. You cannot always expect everything from the people [staff], but … but then you come to the point [the problem of the available staff] again of the staff and I am so disappointed in that, that it’s so bad.” **FM**
C. **Deficiency of staff knowledge**		
	Deficiency of staff knowledge	C1, I: “Do you think there are enough knowledge and skills available along the whole line and well along the line of your colleagues or other disciplines? (interviewer)
		Pa3: Yes, well … They don’t. We ourselves also don’t. No, maybe that sounds a bit weird, but that’s just the way it is. Yes, I mean it’s also very important for yourself.” **LPN**
		C2, I: “But are you saying that knowledge in all areas is not always present?
		Pa4: No … In all areas it’s not. **RLPN**
		I: That is also what others also …
		All: Yes …
		Pa3: Yes, I think so. I think we all lack enough knowledge. **LPN**
		Pa3: We sometimes know more about the computer than we know about that [neuropsychiatric symptoms]. Sometimes yes. You need to have more knowledge about that.” **LPN**
		C3, pa3: “And if someone totally panics because he sees big spiders walking on the wall, then you know …. Oh … that fits the picture of the disease. So, he sees things that are not there. You can panic about that and so yes … as long … if you don’t have that knowledge … then you would think … that man is not well at all. I have to call the physician quickly as he has to go to the hospital.” **LPN**
D. **Inadequate (multidisciplinary) collaboration**		
	Lack of evaluation	D1, pa10: “In the past several years, if someone has a restriction of freedom, that that will usually remain that way. And before it comes up for discussion again or before it gets discussed like is it actually still necessary that someone is restrained, that woman is not going to get up anymore. That you … If no one makes a remark about it, that that sometimes persists longer than necessary.” **BC**
		D2, I: Evaluation of psychotropic drugs, how does that work?
		Pa7: Oftentimes, that doesn’t happen. **NP**
		Pa9: Very little. Well longer than the three months that were allowed, after which it should be evaluated. That was … yeah often too long. **N**
		Pa7: Yes, I think that this needs more attention. What the psychologist also said, we need to address decisions that have been made and evaluate those again, that is missing.” **NP**
	Lack of (multidisciplinary) consultation of key disciplines	D3, pa8: “We are actually never present at such meetings [multidisciplinary consultation]. {…} It would be relevant if we’d be present there. Because we work in the evenings, we work at night, the weekends. We are here such a big part of the time. We are always the ones that get called.” **N**
		D4, pa4: “The physician has really been busy with all medication … to look into it per resident and consult with the family and to stop many medications. {…} I had one resident … they, yes I think that, experimented with him a lot. And then I can feel something about it, I can say something, but they don’t listen to you and then I think [curse] … He went from one medication to the other because it wasn’t working and then I think … Well just stop with it for once. Because maybe it is all counterproductive and eh … I think we should have more of a say in these matters.” **RLPN**
	Lack of multidisciplinary consultations / meetings	D5, pa10: “It is also weird that we have never had a meeting. That is what I am thinking now. Because actually you do … What you do in the evening and at night, is what I do during the day. BC (pa10)
		Pa 8 & 9: Yes. **N**
		Pa10: We have never had a meeting about that.” **BC**
E. **Suboptimal communication**		
	Flawed internal reporting and communication	E1, pa 11: “Well I find the communication very bad among the workers. **FM**
		Pa13: Yes. **FM**
		I: But that is very general, what do you mean?
		Pa111: One does not know what the other does. **FM**
		I: Within the group?
		Pa11: Within the group. Nursing staff … Yes …” **FM**
		E2, Pa13: “I say it often, everything goes well up until the door of the unit. And then the problems start. **FM**
		I: {…}
		Pa13: No, I mean the planning and communication, those I find very bad from that side [of the management], the higher you get in management.” **FM**
		E3, Pa6: I think that the coordination therein is also important. Because hearing this question, I also think how many conversations do I have with relatives? Not that much, but also because I’m assuming that the physician does that or the registered nurse or care staff.” **P**
	Lack of sharing experiences	E4, pa6: “The sad part again is that results are not really shared and it could be so inspiring if you know: ‘Wow, we did that very well together. And someone went from very unhappy and displaced to … enjoying a pleasant gettogether, while that is the thing that provides a good vibe, next time we can do this together too’.” **P**
		E5, pa10: “Especially the old school, they really have a … really a … a culture of wanting to control, they want to have the right touch. And if they need to ask for help, sometimes that is a … that is too much to ask. Or a … Or … One is not so easily inclined to share a problem. They keep it to themselves. And I find that very unfortunate.” **BC**
		E6, pa7: “That we would give more feedback to each other. That we knock our heads together. How is that man or woman doing? And that the behavioral coach is present and maybe the RLPN. I think that could be improved … Well that can be improved.” **NP**
	Unclear communication of changes with family	E7, pa18: “Yes, then the family is not informed … {…}, then it was not clear why it was stopped. Everything was just stopped. Everything can go. But it does not work that way. Because family wants to be informed with every change in medication, why is it stopped, with explanation.” **NA**
		E8, pa7: “Recently a resident or a partner of a resident told me … She said: “I want to be more involved in the decision-making regarding the treatment of my husband. That has never happened in the past years, I was totally ignored in this area.” She was very unsatisfied with this. {…} In principle we do that! The care staff link it back to the family. And if there are decisions with a big impact in terms of medication, then I contact them myself, but it would appear that it has not always happened. In that way I think we leave some loose ends sometimes. I don’t want to say that I always inform everyone, but at least I try to. **NP**
	Communication with relatives is considered time consuming	E9, Pa4: Officially it [resident background information] should be according to the domains [of life]. But there is so little time to … I myself am an RLPN, to fill it in. To start the dialogue with a relative because you have to do it by means of a form. I think that’s … difficult to start that conversation, to plan it or … plan the conversation is not a problem, but … to fill it in you know … You have to talk about all the different domains … It takes an incredible amount of time. So usually we discuss the workplan, we make that and then over time we shortly add the things we have a need for, but it will never be as detailed as it would be according to the domains.” **RLPN**
F. **Disorganization of processes**		
	Unstructured processes	F1, I: “But then you have to evaluate some things.
		Pa7: Yes, and that, that could be improved. I think so, yes. So we actually have to structure that too, shouldn’t we?” **NP**
		F2, pa8: “Also the past period there have been many changes [turnover of physicians] here, which caused the systematics to get a little lost. And eh … What we also said before, you need continuous people to consult with each other and to … to apply policies. {…} with constantly changing staff especially in the ranks of the physicians, you get a very ad hoc approach. And that is how things are, because if someone is present very little or … not present fulltime, either there is someone else all the time, you have to make new agreements every time...” **P**
		F3, pa9: “Yes except we have no clear timespan, how long will you keep trying? {…} People can be willing to keep investigating things, because maybe if we do this, that will have the effect we’re hoping for. We don’t have much clarity about the timespan and sometimes that could be very important. If that doesn’t work than we have to take action quickly, to try to break through the pattern with medication. **P**
		Pa7: Yes, I think that can be more structured too.” **NP**
		F4, pa10: “Yes, there are many good ideas, that is not the problem, but in one way or another it doesn’t come together. That is my feeling.” **BC**
	Ambiguity of division of responsibilities and tasks	F6, pa6: “I think it is important, that they [physician and psychologist] are in a position … in which they can collaborate. So that it is clear, who does which task? Eh … Who is the coordinator? Is the physician the main point of contact in case of NPS or is it the nurse practitioner? Or is it the psychologist? I sometimes find that difficult, I sometimes think who is the captain on that ship?” **P**
		F7, pa6: “I sometimes find it hard in the collaboration with the unit manager, the role of the unit manager and the collaboration. It’s not just the teams, but also what is each other’s role in that way?” **P**
		F8, pa5: “In clinical practice it is found that it is hard to execute [to extract the life history]. Very concrete over time, who executes it? Does the care staff execute it, or does the psychologist do it, the social worker that we have had here for some time, now not anymore? So who will do it?” **P**
		F9, pa7: “The feedback to the RLPN on do you this or shall I do it, sometimes I leave loose ends. **NP**
		F10, pa10: “I don’t know what kind of role you [registered nurses] have exactly. I thought that it was purely medical … the medical area so to say, so an extension of the physician.” **BC**
		F11, pa6: “Well, for example if there is resistance of care staff to … to do certain things or an intervention or people say they will do it and they won’t. Who is going to guide that? Who is responsible then? Of course I can address, but if it’s a motivational problem, well … then it is not up to me to find it out where the problem is located.” **P**
		F12, Pa6: Coaching of the team, really guide them, to pep them up and give them energy, inspire them, yes, I think that’s something for the unit manager to do. **P**
		Pa9: But for that purpose, you see them too little on the unit, the unit managers. I think that is also one of the problems. **N**
		Pa6: The unit managers are too busy with the planning in my opinion. That kind of stuff.{…} While to me, that’s not their primary task. So that part is something they can develop themselves in, together with the multidisciplinary team. And then also the support of the management for the unit manager.” **P**
	Decision-making culturesus	F13, pa1: “Before you have managed to make a change. Everybody thinks something about it and eh …” **UM**
G. **Reactive coping &*****resilience of organization***		
	Difficulty breaking patterns	G1, pa1: “{…} things that are like this for years, that is very hard to break through, to change. That I encounter with everything on this care unit.” **UM**
		G2, pa1: “We have a recreational therapist that is really on the unit. But she is still so busy with actually … well … coordinating volunteers, that is actually what she’s doing at the moment. Yes … she has to change her work routines. But that is very difficult for her. I’m talking about it with her now. But we all have a clear picture of what we want from the care perspective. And that doesn’t change overnight.” **UM**
		G3, pa2: “But also things that have been this way for years like those residents go to drink coffee every morning and then the others stay behind and then I had a big discussion about that with her [occupational therapist]. {…} Those things are so rigid, those residents do this all days, so …” **LPN**
	Concerns relatives on changing practice	G4, pa15: “The nursing staff determines what my husband’s day looks like. **FM**
		Pa11: And you have no say in the matter. **FM**
		Pa15: No … **FM**
		Pa11: You can … you may, but nothing will happen … It will not be addressed.” **FM**
		G5, Pa16: I myself am part of the board [of client representatives] … It doesn’t help much. I actually miss that a bit {…} We are in the middle of the residents, between clients and between the management. It has to have more of a voice in matters. Because they present a plan of care {…}. We only have to read it. Well … Look then it’s already too late. That is too late … {…} Because they already took the decision and the board of resident representatives only has to say if they agree with it, that’s what we’re good for.” **FM**
		G6, pa25: “My husband came here on the unit. He was here at the daycare. Well then it came to pass that he actually had to stay here [on the unit]. So we had a look at one of the units. What they presented us then … It is going to be like this and there will be a fence, the doors will open, people will be able to walk around outside and all those things and more.**FM**
		I: That hasn’t happened yet …
		Pa16: No nothing … **FM**
		Pa11: Up until today not yet. …” **FM**
		G7, pa10: “We do notice in the nursing home, there are a lot of good ideas and a lot of nice developments and eh … Those are actively pursued but it also always kind of slips away.” **BC**
	Responding late to behavior	G8, pa8: “We are actually only called when there is something wrong with the resident. If we just have more information [to help them] … not. **N**
		Pa5: That also happens very often with us. We are being called, I don’t want that anymore actually, only if there are difficult situations, but you should have to chance to get to know the people a bit. And that is actually our main goal, I think the perspective of the person, the goal is also not to reduce people, but to get a complete picture of them.” **P**
		G9, pa5: “There is too much thinking going on or waiting for too long … You’ll get some kind of escalation, an accumulation of behavior. I view all behavior as normal behavior, it’s all … It fits our residents; it is an expression of something. You have to look for the meaning of it. And if you wait too long with that. Only if there is … A last … disruption of the balance in that person of in their environment, then they blow the whistle and that way of thinking and observing, I would like to see that changed.” **P**
		G10, pa3: “Yes well he [psychologist] cannot give the solution immediately, however we can think together, well … how can we prevent this from happening and he can provide us with the tools. And then we are searching for a solution together. Instead of bringing the salt afterwards when the egg is already finished. Then it has already happened.” **LPN**
	Not signaling changes in behavior	G11, pa15: “There should be a regular team on every unit. To ensure that the people who are there know okay … This resident behaves different from yesterday. **FM**
		Pa 14 &16: Yes. **FM**
		Pa15: What we see now … Today it’s that person, tomorrow someone else, but that person doesn’t see that he [the resident] might be different from the day before or from normal. **FM**
		I: If it is not noticed in time …
		Pa15: If it is not noticed in time … or whatever then it is necessary for us to stay on top of things all the time. Because it has to come from us like guys there is something wrong, he’s behaving differently, he is not usually like this.” **FM**
H. **Differences in perception**		
MENTIONED	Expressed differences in perception between colleagues	H1, pa4: “But what I think is disturbing, doesn’t have to be disturbing for her [other LPN] or doesn’t have to be a problem for her [other LPN]. It has something to do with you, as an individual. That is why we have to consult each other. We are all different.” **RLPN**
		H2, pa4: “Every physician has their own working method. Their own way of thinking. And their own vision on that [psychotropic drugs].” **RLPN**
		H3, pa17: “Well some [physicians] prescribe a bit faster than others. And you’ll respond quickly to what somebody says. Because the care staff calls and now I’m saying this without nuance. But “oh that resident is agitated so can’t we give her a pill?” Some will say yes that is possible and will prescribe so to say and others will say but when does she get agitated and what happened before …” **UM**
		H4, pa6: “I think those [restrictions of freedom of the resident] are being evaluated by the physician in the rounds, monthly. That’s not something that’s discussed multidisciplinary … **P**
		pa7: “If I’m honest, I have never experienced that [evaluation of restrictions of freedom of the resident] before.” N**P**
		H5, pa18: “{…} multiple residents already went to bed with clothing over their pajama. I was thinking, what is this?! First getting them out again … Yeah … Because I couldn’t find them [in the living room]. **NA**
		Pa17: You could also just let them sleep. **UM**
		Pa18: Yeah, but yeah … Then they would have done it on their own. That felt very wrong. For me … **NA**
		Pa20: I hadn’t undressed them again. **LPN**
		Pa18: Yeah then my colleague will come the next day saying what a mess has pa18 left behind. **NA**
		Pa20: Yeah, well … too bad.” LPN
NOTICED	Observed differences in perception between focus group participants	*Difference in perspective on participation of different disciplines in multidisciplinary consultation.* H5, pa5: “People are broadly discussed in the multidisciplinary meetings. There we address what they need … {…} What would be good interventions, fitting for that person. So then we have a much broader context than … that is where we talk about someone. Of course not everyone is present. For example, you [registered nurses] do not have anything to do with that.” **P**
		**Later on in the same focus group**
		H6, pa8: “We are actually never present at such meetings [multidisciplinary consultation]. {…} It would be relevant if we’d be present there. Because we work in the evenings, we work at night, the weekends. We are here such a big part of the time. We are always the ones that get called.” **N**
		*Multidisciplinary consultation (structuring of consultation necessary or not necessary difference in opinion)*
		H7, pa17: “… Moments to evaluate usually happen in a very small setting. Only those who … A multidisciplinary consultation always sounds so big. But then there are the evaluation moments and those can be planned at any opportunity, whenever it’s necessary. So that’s what we do. That doesn’t need to be structured.” **UM**
		**Discussion in another focus group:**
		H8, pa9: “I think those [restrictions of freedom] are being evaluated by the physician in the ward round. And monthly. That is not something that is being discussed multidisciplinary, but I think that the physician, that is being discussed with the physician. **P**
		Pa10: Maybe that will improve now? **BC**
		Pa7: I have never experienced that to be very honest. I think that too can be improved.” **NP**
		*Discrepancy between what nursing staff does and what the NP thinks that happens.* H9, pa7: “Recently a resident or a partner of a resident told me … She said: “I want to be more involved in the decision-making regarding the treatment of my husband. That has never happened in the past years, I was totally ignored in this area.” She was very unsatisfied with this. So in that way I have learned from this case to maybe … In principle we do that! The care staff links it back to the family. And if it are decisions with a big impact in terms of medication, then I contact them myself, but it would appear that it has not always happened. In that way I think we leave some loose ends sometimes. I don’t want to say that I always inform everyone, but at least I try to. **NP**
		**Nursing staff in another focus group:**
		H10, pa18: “Yes, then the family is not informed … {…}, then it was not clear why it was stopped. Everything was just stopped. Everything can go. But it does not work that way. Because family wants to be informed with every change in medication, why is it stopped, with explanation.” **NA**
		*Complaints*
		H11, pa18: “The family sometimes has some complaints to us, that there is a lot of different staff again, that happens quite often actually. **NA**
		Pa17: Yes, well that is the only complaint they have. **UM**
		**While in the focus group of relatives:**
		H12, Pa15: The nursing staff determines what my husband’s day looks like. **FM**
		Pa11: And there is nothing you can do about that. **FM**
		Pa15: No … **FM**
		Pa11: You can … You are allowed, but nothing … Nothing is done about it.” **FM**

## Abbreviations

BC: Behavioral coach
LPN: Licensed practical nurse
NA: Nurse assistant
NHS: The english national institute for health and clinical excellence
np: nurse practitioner
NPS: Neuropsychiatric symptoms
P: Psychologist
RID: Reduction of Inappropriate psychotropic Drug use in nursing home patients with dementia
RLPN: LPN responsible for the coordination of care for individual residents
RN: Registered nurses
Themes: Themes of barriers
UM: Unit manager
UMCG: University medical center groningen
