# Supplementary material for: The construction of a conceptual framework explaining the relation between barriers to change of management of neuropsychiatric symptoms in nursing homes: a qualitative study using focus groups
Source: BMC Geriatr. 2020 May 6;20:163. doi: 10.1186/s12877-020-01569-w (PMC7201759; doi:10.1186/s12877-020-01569-w)
Supplement: Supplementary file 1 — Additional file 1: Supplement A. Detailed applied methodology following the consolidated criteria for reporting qualitative studies (COREQ) 32-item checklist [file 12877_2020_1569_MOESM1_ESM.docx]

**Supplement A – Detailed applied methodology following the consolidated criteria for reporting qualitative studies (COREQ) 32-item checklist**

| **No.** | **Item** | **Guide questions/description** | **Our research** |
| --- | --- | --- | --- |
| **Domain 1: research team and reflexivity** | | | |
| 1. | Interviewer/facilitator | Which authors conducted the interview? | LP (de Hoven, Delfzijl) |
| 2. | Credentials | What were the researcher’s credentials? | LP: MSc.  CT: MSc.  KV: MSc.  DG: MSc.  AP: BSc.  SUZ: MSc.  MS: MSc. |
| 3. | Occupation | What was their occupation at the time of the study? | LP: Psychologist at another location of the same nursing home.  CT: Medical doctor  KV: Research assistant  DG: Researcher  AP: Registered nurse  SUZ: Elderly care physician, head researcher  MS: Elderly care physician, researcher |
| 4. | Gender | Was the researcher male or female? | LP: Female  CT: Female  KV: Female  DG: Female  AP: Female  SUZ: Male  MS: Male |
| 5. | Experience and training | What experience or training did the researcher have? | LP: Master’s degree in psychology.  CT: Master’s degree in medicine  KV: Master’s degree in psychology, minor experience in qualitative research  DG: Master’s degree in psychology, extensive experience in qualitative research  AP: Bachelor’s degree in nursing, extensive experience in qualitative research  SUZ: Master’s degree in Medicine  MS: Master’s degree in Medicine |
| *Relationship with participants* | | | |
| 6. | Relationship established | Was a relationship established prior to study commencement? | The researcher CT communicated with the unit managers about participation in the project. Furthermore, she interacted with both nurse practitioner, psychologists and LPN/RLPN for the data collection via questionnaires before the focus groups were conducted. |
| 7. | Participant knowledge of the interviewer | What did the participant know about the researcher? e.g. personal goals, reasons for doing the research. | Before every focus group the participants were informed of the reasons for doing the research as part of the pilot project for the RID study.  In addition, the occupation of the moderator (LP) and researcher present was disclosed before the start of every focus group. |
| 8. | Interviewer characteristics | What characteristics were reported about the interviewer/facilitator? e.g. Bias, assumptions, reasons and interests in the research topic. | It was orally stated prior to every focus group that the researchers had no conflict of interest with the outcome of the conducted qualitative research. |
|  |  |  |  |
| **Domain 2: study design** | | | |
| *Theoretical framework* | | | |
| 9. | Methodological orientation and Theory | What methodological orientation was stated to underpin the study? e.g. grounded theory, discourse analysis, ethnography, phenomenology, content analysis. | Thematic analysis according to Braun & Clarke [21] |
| *Participant selection* | | | |
| 10. | Sampling | How were participants selected? e.g. purposive, convenience, consecutive, snowball. | Participants were selected by the unit managers and approached by the researchers. During this selection procedure special attention was paid to select people with the ability to voice an honest opinion for the focus group with nurses as well as the group of relatives. |
| 11. | Method of approach | How were participants approached? e.g. face-to-face, telephone, mail, email. | Staff of the nursing home was approached face-to-face for participation by either the unit manager or researcher (SUZ / CT). Relatives were approached via mail by researcher CT. |
| 12. | Sample size | How many participants were in the study? | Total participants: 23  Focus group 1 – Nursing staff traditional care unit: 4  Focus group 2 – Treatment staff: 7  Focus group 3 – Relatives of residents: 6  Focus group 4 – Nursing staff small-scale living: 6 |
| 13. | Non-participation | How many participants refused to participate or dropped out? Reasons? | We mailed invitation letters to ten relatives, two relatives stated that the time and date of the focus group didn’t fit their schedule. From two others we didn’t receive a reply. |
| *Setting* | | | |
| 14. | Setting of data collection | Where was the data collected? e.g. home, clinic, workplace | All focus groups were carried out in a multifunctional room of the local nursing home. |
| 15. | Presence of non-participants | Was anyone else present besides the participants and researchers? | No |
| 16. | Description of sample | What are the important characteristics of the sample? E.g. demographic data, date. | Gender, occupation and relation of relatives to the residents. |
| *Data collection* | | | |
| 17. | Interview guide | Were questions, prompts, guides provided by the authors? Was it pilot tested? | There was a topic list with topics, questions and prompts provided to the interviewer. |
| 18. | Repeat interviews | Were repeat interviews carried out? If yes, how many? | No, focus groups were at a single point in time. |
| 19. | Audio/visual recording | Did the research use audio or visual recording to collect the data? | All interviews were audio-taped and transcribed ad verbatim (by CvT). |
| 20. | Field notes | Were field notes made during and/or after the interview or focus group? | No |
| 21. | Duration | What was the duration of the interviews? | The mean duration of the focus groups was 90 minutes, with a range from 84 to 115 minutes. |
| 22. | Data saturation | Was data saturation discussed? | No |
| 23. | Transcripts returned | Were transcripts returned to participants for comment and/or correction? | No |
| **Domain 3: analysis and findings**  *Data analysis* | | | |
| 24. | Number of data coders | How many data coders coded the data? | CT, KV |
| 25. | Description of the coding tree | Did authors provide a description of the coding tree? | The description of the coding can be obtained through the authors. |
| 26. | Derivation of themes | Were themes identified in advance or derived from the data? | Themes were derived from the data. |
| 27. | Software | What software, if applicable, was used to manage the data? | Analysis was done with Atlas.ti 7.5.10 (Atlas.ti Scientific Software development GmbH, Berlin, Germany(. |
| 28. | Participant checking | Did participants provide feedback on the findings? | Yes, participants received a PowerPoint presentation with the findings from the focus groups, and they were invited to provide feedback. |
| *Reporting* | | | |
| 29. | Quotations presented | Were participant quotations presented to illustrate the themes/findings? Was each quotation identified? e.g. participant number. | Yes, see Results section and Table 1. |
| 30. | Data and findings consistent | Was there consistency between the data presented and the findings? | Yes |
| 31. | Clarity of major themes | Were major themes clearly presented in the findings? | Yes, see Results section and Figure 2 |
| 32. | Clarity of minor themes | Is there a description of diverse cases or discussion of minor themes? | Yes, see Table 1 |
